# Methylation of *BDNF* and *SLC6A4* Gene Promoters in Brazilian Patients With Temporal Lobe Epilepsy Presenting or Not Psychiatric Comorbidities

**DOI:** 10.3389/fnint.2021.764742

**Published:** 2021-11-29

**Authors:** Isabel Cristina Bandeira, Lucas Giombelli, Isabel Cristina Werlang, Ana Lucia Abujamra, Thais Leite Secchi, Rosane Brondani, José Augusto Bragatti, Jorge Wladimir Junqueira Bizzi, Sandra Leistner-Segal, Marino Muxfeldt Bianchin

**Affiliations:** ^1^Graduate Program in Medicine: Medical Sciences, Universidade Federal do Rio Grande do Sul, Porto Alegre, Brazil; ^2^Basic Research and Advanced Investigations in Neurosciences, Experimental Research Center, Hospital de Clínicas de Porto Alegre, Porto Alegre, Brazil; ^3^Division of Neurology, Hospital de Clínicas de Porto Alegre, Porto Alegre, Brazil; ^4^Division of Neurosurgery, Hospital de Clínicas de Porto Alegre, Porto Alegre, Brazil; ^5^Medical Genetics Service, Hospital de Clínicas de Porto Alegre, Porto Alegre, Brazil; ^6^Centro de Tratamento de Epilepsia Refratária (CETER), Hospital de Clínicas de Porto Alegre, Porto Alegre, Brazil

**Keywords:** methylation, psychiatric comorbidities, depression, *BDNF*, serotonin, neurotrophins

## Abstract

The relationship between epilepsy and psychiatric comorbidities has been recognized for centuries, but its pathophysiological mechanisms are still misunderstood. It is biologically plausible that genetic or epigenetic variations in genes that codify important neurotransmitters involved in epilepsy as well as in psychiatric disorders may influence the development of the latter in patients with epilepsy. However, this possibility remains poorly investigated. The aim of this study was to evaluate the methylation profile of the *BDNF* and *SLC6A4*, two genes importantly involved in neuroplasticity, in patients with temporal lobe epilepsy (TLE) regarding the development or not of psychiatric comorbidities. One hundred and thirty-nine patients with TLE, 90 females and 45 males, were included in the study. The mean age of patients was 44.0 (+12.0) years, and mean duration of epilepsy was 25.7 (+13.3) years. The Structured Clinical Interview for DSM-IV shows that 83 patients (59.7%) had neuropsychiatric disorders and 56 (40.3%) showed no psychiatric comorbidity. Mood disorders were the most common psychiatric disorder observed, being present in 64 (46.0%) of all 139 patients. Thirty-three (23.7%) patients showed anxiety disorders, 10 (7.2%) patients showed history of psychosis and 8 (5.8%) patients showed history of alcohol//drug abuse. Considering all 139 patients, 18 (12.9%) demonstrated methylation of the promoter region of both *BDNF* and *SLC6A4* genes. A significant decreased methylation profile was observed only in TLE patients with mood disorders when compared with TLE patients without a history of mood disorders (O.R. = 3.45; 95% C.I. = 1.08–11.11; *p* = 0.04). A sub-analysis showed that TLE patients with major depressive disorder mostly account for this result (O.R. = 7.20; 95% C.I. = 1.01–56.16; *p* = 0.042). A logistic regression analysis showed that the independent factors associated with a history of depression in our TLE patients was female sex (O.R. = 2.30; 95% C.I. = 1.02–5.18; *p* = 0.044), not controlled seizures (O.R. = 2.51; 95% C.I. = 1.16–5.41; *p* = 0.019) and decreased methylation in *BDNF* and *SLC6A4* genes (O.R. = 5.32; 95% C.I. = 1.14–25.00; *p* = 0.033). Our results suggest that *BDNF* or *SLC6A4* genes profile methylation is independently associated with depressive disorders in patients with epilepsy. Further studies are necessary to clarify these matters.

## Introduction

Epilepsy is one of the most prevalent neurological diseases, affecting people of all ages. According the World Health Organization, about 50 million people worldwide have been diagnosed with epilepsy (Ngugi et al., 2010; World Health Organization, 2015). Because of factors such as high prevalence, severity, morbidity, and socioeconomic impact, scientific research in the field of epileptology became a priority in public health policies (Li and Sander, 2003; Thurman et al., 2011).

The relationship between epilepsy and psychiatric disorders has been recognized for centuries. However, the wide spectrum of neuropsychiatric comorbidities and its extension have been more investigated only over the last decades. The impact of these comorbidities on behavior related to seeking help, seizure control and quality of life suggests that prompt detection and treatment of these problems is crucial. Therefore, it is important to recognize accurately the occurring neuropsychiatric condition in people with epilepsy in order to provide appropriate management of these patients (Devinsky, 2003; Agrawal and Govender, 2011; Kanner, 2017).

The prevalence of psychiatric disorders is higher in people diagnosed with epilepsy when compared to the general population. In patients with epilepsy, depression may occur in about 30% of patients, anxiety disorders in 10–25%, and psychosis in 2–7% (Gaitatzis et al., 2004). These patients are three times more likely to commit suicide than the general population (Bell et al., 2009). However, most studies focus on the relationship between epilepsy and depression and, consequently, there is a deficit of epidemiological data regarding the association between epilepsy and the remaining psychiatric disorders (Devinsky, 2003; Gaitatzis et al., 2004, 2012; Bragatti et al., 2011, 2014; Schenkel et al., 2012).

Epigenetic mechanisms are influenced by environmental factors and play a role in several aspects of neuronal function, from embryogenesis and early brain development to tissue-specific gene expression and global gene silencing. It is therefore plausible that an epigenetic dysregulation may play a significant role in brain disorders such as neuropsychiatric disorders or epilepsy (Levenson and Sweatt, 2005; Tsankova et al., 2007; Graff et al., 2011; Hauser et al., 2018). To date, DNA methylation is one of the best understood epigenetic mechanisms. It occurs primarily in DNA sequences rich in cytosine residues adjacent to guanine residues, known as CpG sites. Alterations in DNA methylation have been reported in temporal lobe epilepsy (TLE) patients, with several genome-wide studies supporting evidence that epilepsy progression is accompanied by many changes in the methylome (Kobow et al., 2013; Debski et al., 2016).

BDNF plays an important role in the growth and differentiation of new neurons and synapses, as well as in the survival of existing neurons of the central and peripheral nervous system (Fargali et al., 2012; Ichim et al., 2012). Evidence suggests a potential contribution of BDNF and its receptor, TrkB, in the pathophysiology of epilepsy. *In vivo* and *in vitro* studies have shown that BDNF levels and activity are increased during epileptogenesis (Scharfman, 2002, 2005). Moreover, the reduction of TrkB levels in the hippocampus of adult rats decreases the occurrence of seizures, suggesting that TrkB could be a target for therapeutic intervention in epilepsy (Kotloski and McNamara, 2010; Heinrich et al., 2011).

Serotonin is an important neurotransmitter in the central nervous system (CNS), playing several roles in brain development and in neuroplasticity. It also affects the balance between excitatory and inhibitory neural pathways and thus it might be involved in various physiological and pathological processes within the brain, including epilepsy (Theodore, 2003; Jamali et al., 2006; Muller and Homberg, 2015; Lv and Liu, 2017). The role of *SLC6A4* variants, the gene that encodes the serotonin transporter, has been studied regarding the etiology or neuropsychiatric comorbidities in epilepsy. Additionally, clinical studies involving image and pharmacology suggest that changes in serotonergic transmission may play an important role in TLE and in the comorbidities associated with this condition (Manna et al., 2007; Stefulj et al., 2010; Schenkel et al., 2011; Córdoba et al., 2012; Martinez et al., 2013). *SLC6A4* is of particular interest regarding epigenetic changes. It is believed that the methylation of *SLC6A4* may contribute to vulnerability to neuropsychiatric disorders (Beach et al., 2010, 2011; Devlin et al., 2010; Koenen et al., 2011). SLC6A4 is an integral membrane protein, primarily in the central and peripheral nervous systems, and carries serotonin (5-HT) from synaptic spaces into presynaptic neurons, regulating the emotional aspects of behavior (Meyer-Lindenberg, 2009).

Because of the high prevalence of psychiatric conditions in patients with epilepsy, it is plausible that this association might occur due to pathogenic mechanisms common to both disorders. If this is true, one can also expect that a better understanding of these mechanisms at the molecular level may eventually bring new knowledge regarding the inception of epilepsy or psychiatric disorders and the association between both. Moreover, it may also allow for the development of alternative therapies for these diseases (Gaitatzis et al., 2004; Tsankova et al., 2007). The purpose of this study was to evaluate the role of methylation events in the *BDNF* and *SLC6A4* promoter regions in the development of neuropsychiatric comorbidities in patients with TLE. We believe that this study may contribute to understanding the genesis of psychiatric comorbidities in patients with epilepsy, particularly in patients with TLE.

## Materials and Methods

### Patients and Samples

We selected 150 consecutive patients with TLE, selected from the Epilepsy Outpatient Clinic of Hospital de Clínicas de Porto Alegre (HCPA). Inclusion criteria were based on the ILAE’s electroclinical classification (Commission on Classification Terminology of the International League Against Epilepsy) and neuroimaging results (Berg et al., 2010; Fisher et al., 2014, 2017; Scheffer et al., 2017). Eleven patients were removed from the study due to incomplete data and 139 patients were included. The Ethics Committee of our institution, in accordance with the Declaration of Helsinki, approved the study and all subjects provided written informed consent to participate.

Genomic DNA was extracted using the salting-out method previously described by Miller et al. (1988). For the methylation profile analysis, the conversion procedure with sodium bisulfite was performed in the DNA samples. This process consists in the conversion of unmethylated cytosines to uracil. If the cytosine is methylated this change will not occur. Bisulfite conversion was carried out by using bisulfite EpiTect Kit (Qiagen, Hilden, Germany) according to the manufacturer’s instructions.

### HRM Analysis

Methylation profile analysis was performed using High Resolution Melting method on the StepOne™ Real-Time PCR System equipment (Applied Biosystems^®^). We accessed two promoter regions of the *BDNF* gene, in exons I and IV. The primers used were previously described by D’Addario et al. (2012) and Ikegame et al. (2013). The *SLC6A4* promoter region was accessed with primers described by Kim et al. (2013). As controls, we used Cells-to-CpG Methylated and Unmethylated gDNA Control Kit (Applied Biosystems^®^). To create the range of methylated and unmethylated dilutions, the controls were mixed to obtain the following ratios of methylation: 0, 25, 75, and 100%. Standard curves with known methylation ratios were included in each assay and were used to infer the methylation ratio of each sample.

All analyses were performed according to the following conditions: holding step (95°C for 10 min), 40 cycles of 95°C for 15 s and 60°C for 1 min, followed by an HRM step of 95°C for 10 s, 60°C for 1 min, 95°C for 15 s, and 60°C for 15 s, with continuous acquisition at every 0.2°C. HRM mix was performed in a final volume of 10 μL, containing 5 μL of MeltDoctor mastermix (Applied Biosystems), 5 pmol of each primer and 1 μL (approximately 20 ng/μL) of bisulfite modified DNA sample. Each reaction was performed in duplicate. After amplification by HRM, the data were analyzed using High Resolution Melting software version 3. All samples of interest were purified and sequenced by Sanger’s method to confirm our results. The sequences were aligned using the BioEdit program’s *CLUSTALW* algorithm, manually inspected.

### Psychiatric Evaluation

All patients were assessed by means of the Structured Clinical Interview for DSM-IV (SCID) (First et al., 2001), divided into six modules, for the detection of one or more lifelong diagnoses from the Axis I Diagnostic and Statistical Manual, fourth edition (DSM-IV) (American Psychiatric Association, 2000). Patients were classified according to the presence or absence of psychiatric comorbidities, in mood disorder, psychosis disorders, anxiety disorder, and alcohol/drug abuse disorder, and included in the TLE group of patients with psychiatric comorbidity (SCID positive). These patients were compared to the patients without any psychiatric comorbidities (SCID negative) for clinical and methylation status differences. Also, regarding to the methylation status, TLE patients with mood disorders were compared with patients without mood disorders, patients with anxiety disorders were compared with patients without anxiety disorders, patients with history of psychosis where compared with patients without history of psychosis and patients with history of alcohol or drug abuse were compared with patients without history of alcohol or drug abuse. Analysis of sub-groups were also performed for patients with mood disorders. Those patients were also compared with patients without history of any psychiatric disorder regarding methylation profile.

### Statistical Analysis

We assessed the statistical differences between TLE patients with or without psychiatric comorbidities and the methylation status of the *BDNF* and *SLC6A4* gene promoters. Patient and control groups were studied, evaluating clinical variables related to the epileptogenic process and psychiatric comorbidities. The variables directly related to epilepsy were gender, age, age of epilepsy onset, duration of epilepsy, seizure control, and history of depression or epilepsy in the family, neuroimaging findings, and medicines in use. Good seizure control was defined as no seizures at last 1 year before the inclusion in the study. Data were analyzed statistically by the Fisher’s Exact Test for qualitative variables. For quantitative variables, the independent *T*-test with Levene’s test for equality of variances was used. Qualitative variables are expressed as percentage with odds ratio (95% C.I.). Quantitative variables are expressed as mean (±SD). Unconditional logistic regression was used to control for independence of the associations observed. Variables with *p* ≤ 0.20 were included in the logistic regression model used. All statistical analyses were carried out with the SPSS statistical package for Windows (SPSS Inc., Chicago, IL, United States). Results were significant if *p* was lower than 0.05.

## Results

**FIGURE 1:**
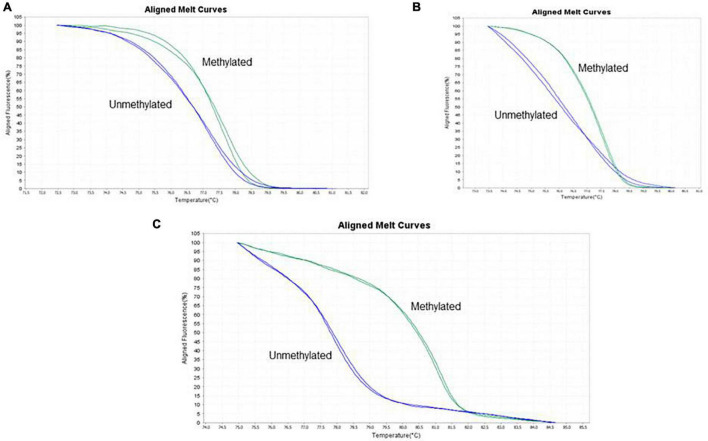
The screen printed shows the interpretation of the raw data generated by the StepOne equipment after the HRM assay is processed, having been generated by the High Resolution Melting software. The melting of a DNA fragment consists in successive dissociations and the melting temperature differs depending on the GC content in each sequence. Melting curves are aligned according to the fluorescence level established for each methylation parameter analyzed. The figure shows alignment of methylation control curves with patient samples. **(A)** BDNF exon 1 promoter region, **(B)** BDNF exon IV promoter region, and **(C)** SLC6A4 promoter region. Each image shows one sample comparing to one control showing the similarity of the aligned curves; the unmethylated curve ranged from 0 to 25% and for the methylated curve from 75 to 100%.

Figure 1 shows the methylation patterns used to determine the methylation status of each TLE patient. Out of 139 TLE patients, 18 (12.9%) were methylated at the promoter region of the *BDNF* and *SLC6A4* genes, and 121 (87.1%) were non-methylated.

Table 1 shows the main clinical features of the patients who completed the study. The patients in the TLE group consisted of 90 females (64.7%) and 45 males (32.4%), with a mean age of 44.0 (+12.0) years. Mean duration of epilepsy was of 25.7 (+13.3) years. The SCID shows that 83 patients (59.7%) had at least one neuropsychiatric disorder (TLE with psychiatric comorbidity group) and 56 (40.3%) showed no psychiatric comorbidity (TLE without psychiatric comorbidity group) for SCID interview (Table 1). Table 2 shows all psychiatric diagnosis in our cohort. Mood disorders were the most common psychiatric disorder observed, being present in 64 (46.0%) of all 139 patients. Thirty-three (23.7%) patients showed anxiety disorders, 10 (7.2%) patients showed history of psychosis, and 8 (5.8%) patients showed history of alcohol/drug abuse. Fifty-one (36.7%) patients presented with more than one type of psychiatric comorbidity. Twenty-four patients had concomitant mood and anxiety disorders. Five patients had concomitant mood disorders and history of psychosis. Two patients had concomitant psychosis and anxiety disorders. Four patients had concomitant mood disorder and alcohol/drug abuse. Two patients had concomitant alcohol/drug abuse an anxiety disorders. Fourteen patients showed more than one type of anxiety disorder.

**TABLE 1:** Clinical variables of TLE patients.

Variables	TLE (*n* = 139)
Mean age of patients (years ± SD)	43.99 (12.05)
Epilepsy age onset (years ± SD)	18.40 (14.60)
Mean duration of epilepsy (years ± SD)	25.72 (13.28)
**Sex**	
Male	49(35.3%)
Female	90(64.7%)
**Family history of epilepsy**	
Negative	85(61.2%)
Positive	54(38.8%)
**Family history of psychiatric disorders**	
Negative	84(60.4%)
Positive	55(39.6%)
**Seizure control**	
Controlled	60(43.2%)
Not controlled	79(56.8%)
**SCID IV**	
Positive	83(59.7%)
Negative	56(40.3%)
**Antiepileptic drugs**	
Monotherapy	71(51.1%)
Polytherapy	68(48.9%)
**Benzodiazepine use**	
No	114(82.0%)
Yes	25(18.0%)
**Psychotropic drugs**	
No	111(79.9%)
Antidepressants	23(16.5%)
Association	05(03.6%)

*TLE, temporal lobe epilepsy.*

**TABLE 2:** All psychiatric disorders in TLE patients.

Variables	TLE patients with psychiatric comorbidities – *n* = 83 (100%)
Mood disorders	64(77.2%)
Major depressive disorder	37(44.6%)
Past depressive episode	11(13.3%)
Dysthymic disorder	09(10.9%)
Bipolar disorder	04(04.8%)
Past maniac episode	02(02.4%)
Cyclothymic disorder	01(01.2%)
Anxiety disorders	33(39.8%)
Generalized anxiety disorder	14(16.9%)
Panic disorder	05(06.0%)
Post-traumatic stress disorder	05(06.0%)
Panic disorder with agoraphobia	04(04.8%)
Specific phobia	03(03.6%)
Obsessive compulsive disorder	02(02.4%)
Psychosis	10(12.1%)
Alcohol/drug abuse	08(09.6%)

*TLE, temporal lobe epilepsy.*

Table 3 shows the main clinical features of our cohort divided according to the methylation status of the promoter region of the genes studied. No association was found between methylation status and the main clinical characteristics of the patients. Specifically, no differences were observed for age, age of epilepsy onset, mean duration of epilepsy, gender, family history of epilepsy or family history of psychiatric comorbidities, use of benzodiazepines, number of antiepileptic drugs in use and use of psychotropic drugs. Sixty-three patients (45.3%) showed normal neuroimaging. Neuroimaging was abnormal in 76 (54.7%) patients. Neuroimaging suggestive of hippocampal sclerosis was observed in 40 patients (28.8%), gliosis in 25 (17.9%), and other abnormalities (atrophy, cystic lesions) in 11 (7.91%). Neuroimaging findings were not associated with methylation profile of patients. In this study, we observed a decreased methylation profile in TLE patients with mood disorders. Considering only the 18 patients with methylated promoter regions, 14 (77.8%) patients did not present mood disorder versus 04 (22.2%) patients who presented with mood disorders, a difference which was statistically significant (O.R. = 3.45; 95% C.I. = 1.08–11.11; *p* = 0.04). This result was mainly due to major depressive disorder (MDD) and past history of depression and it was not related to other types of mood disorders like dysthymic disorder, bipolar disorder, or cyclothymic disorder. Because of these finding, we further analyzed the group of patients with mood disorders. In Table 4 the types of mood disorders are presented according to methylation profile. Life history of MDD was observed in 37 patients and most (36 patients, 97.3%) showed a lower methylated profile (O.R. = 7.20; 95% C.I. = 1.01–56.16; *p* = 0.042). We also combined in one group the patients with major depression disorder and patients with a past depressive episode in life. These were the most common and similar type of mood disorders in our TLE patients, and this group was evaluated separately. This analysis also showed that most patients with methylated promoters had no history of depressive disorders (O.R. = 4.91; 95% C.I. = 1.08–22.33; *p* = 0.032) (Table 4). Moreover, we did some of the same analyses as above, but comparing methylation profile in patients with mood disorder, MDD and past depressive episode in life versus methylation profile in those 56 TLE patients that had no history of psychiatric comorbidity and showed all negative results in SCID. This was a control, done to assure that patients with TLE and history of depression were compared with TLE patients without history of any psychiatric comorbidity. Two (4.2%) out of the forty eight TLE patients with MDD or past depressive episode showed methylation in the gene studied versus 09 (16.1%) out of the 56 TLE patients without any psychiatric comorbidity, a not significant difference (O.R = 4.04; 95% C.I. = 0.90–21.49; *p* = 0.060). However, TLE patients with MDD showed significantly lower methylation in the studied genes than TLE patients with no psychiatric comorbidity (O.R. = 6.89; 95% C.I. = 1.01–56.92; *p* = 0.047).

**TABLE 3:** Clinical variables according with *SLC6A4* and *BDNF* methylation.

Variables	Non-methylated *n* = 121 (87.05%)	Methylated *n* = 18 (12.9%)	O.R. (95% C.I.)	*p*-Value
Mean age (years ± SD)	43.89 (12.50)	44.67 (8.64)	–	0.74
Epilepsy age onset (years ± SD)	17.98 (14.62)	21.19 (14.60)	–	0.39
Mean epilepsy time (years ± SD)	26.01 (12.88)	23.69 (15.99)	–	0.49
**Neuroimaging**				
Normal	56 (46.3%)	07 (38.9%)		
Abnormal	65 (53.7%)	11 (61.1%)	0.74 (0.27–2.03)	0.62
**Mood disorders**				
No	61 (50.4%)	14 (77.8%)		
Yes	60 (49.6%)	04 (22.2%)	3.45 (1.08–11.11)	0.04*
**Anxiety disorders**				
No	91 (75.2%)	15 (83.3%)		
Yes	30 (24.8%)	03 (16.7%)	0.61 (0.16–2.24)	0.56
**Psychosis**				
No	112 (92.6%)	17 (94.4%)		
Yes	09 (07.4%)	01 (05.6%)	0.73 (0.09–6.10)	1.00
**Alcohol/drug abuse**				
No	114 (94.2%)	17 (94.4%)		
Yes	07 (05.8%)	01 (05.6%)	0.96 (0.11–8.28)	1.00

TLE, temporal lobe epilepsy; O.R., odds ratio; C.I., confidence interval. *Significant. Bold values* means “statistically significant”.

**TABLE 4:** Mood disorder and antidepressive use according with methylation.

Variable	Non-methylated *n* = 121 (87.1%)	Methylated *n* = 18 (12.9%)	O.R. (95% C.I.)	*p*-Value
**Mood disorders**				
No	61 (50.4%)	14 (77.8%)		
Yes	60 (49.6%)	04 (22.2%)	3.45 (1.08–11.11)	0.04*
**Major depressive disorder**				
No	85 (70.2%)	17 (94.4%)		
Yes	36 (29.8%)	01 (05.6%)	7.20 (1.01–56.16)	0.042*
**Life history of depression**				
No	75 (62.0%)	16 (88.9%)		
Yes	46 (38.0%)	02 (11.1%)	4.91 (1.07–22.33)	0.032*
**Use of antidepressives**				
No	98 (81.0%)	13 (72.2%)		
Yes	23 (19.0%)	05 (27.8%)	0.61 (0.20–1.88)	0.362

TLE, temporal lobe epilepsy; O.R., odds ratio; C.I., confidence interval. *Significant. Bold values* means “statistically significant”.

Table 5 shows variables according to history of depression (patients with MDD plus patients with a history of a past depressive episode), compared with patients with no depression. Females showed more history of depression, however this finding was not significant in the univariate analysis (*p* = 0.092). History of depression was associated with poor seizure control and a lower level of methylation in the genes evaluated. Variables in Table 5 that showed *p* ≤ 0.20 were included in logistic regression model to examine the independence of the associations observed. This analysis is presented in Table 6. In our study, variables independently associated with history of depression were female gender (O.R. = 2.30; 95% C.I. = 1.02–5.18; *p* = 0.044), poorly controlled seizures (O.R. = 2.51; 95% C.I. = 1.16–5.41; *p* = 0.019), and low methylation profile in the promoter region of the *BDNF* and *SLC6A4* genes (O.R. = 5.32; 95% C.I. = 1.14–25.00; *p* = 0.033).

**TABLE 5:** Variables according to life history of depression in TLE patients.

Variables	Depression *n* = 48 (34.5%)	No depression *n* = 91 (65.5%)	O.R. (95% C.I.)	*p*-Value
Mean age (years ± SD)	44.19 (11.53)	43.89 (12.38)	–	0.891
Epilepsy age onset (years ± SD)	19.15 (14.34)	18.00 (14.80)	–	0.661
Mean epilepsy time (years ± SD)	25.21 (13.45)	25.98 (13.26)	–	0.745
**Sex**				
Male	12 (25.0%)	37 (40.7%)		
Female	36 (75.0%)	54 (59.3%)	2.06 (0.95–4.47)	0.092
Fam. hist. of epilepsy				
Negative	27 (56.3%)	58 (63.7%)		
Positive	21 (43.7%)	33 (36.3%)	0.73 (0.36–1.50)	0.465
**Fam. hist. of psychiatric disorders**				
Negative	26 (54.2%)	58 (63.7%)		
Positive	22 (45.8%)	33 (36.3%)	0.67 (0.33–1.37)	0.281
**Seizure control**				
Controlled	15 (31.3%)	45 (49.5%)		
Not controlled	33 (68.7%)	46 (50.5%)	2.13 (1.03–4.55)	0.048*
**Methylation**				
Methylated	02 (04.2%)	16 (17.6%)		
Non-methylated	46 (95.8%)	75 (82.4%)	4.91 (1.07–22.33)	0.032*

TLE, temporal lobe epilepsy; O.R., odds ratio; C.I., confidence interval; Fam. hist., family history. *Significant. Bold values* means “statistically significant”.

**TABLE 6:** Variables independently associated with a life history of depression.

Variables	O.R.	Crude	*p*	O.R.	Adjusted	*p*
		95% C.I.			95% C.I.	
Female	2.06	(0.95–4.47)	0.092	2.30	(1.02–5.18)	0.044*
Not-controlled seizure	2.13	(1.03–4.55)	0.048*	2.51	(1.16–5.41)	0.019*
Non-methylated	4.91	(1.07–22.33)	0.032*	5.32	(1.14–25.00)	0.033*

TLE, temporal lobe epilepsy; O.R., odds ratio; C.I., confidence interval. *Significant. Bold values* means “statistically significant”.

## Discussion

In our study, TLE patients that showed history of depression also showed lower levels of methylation in *BDNF* or *SLC6A4* genes. Although we were not able to find studied about gene methylation in psychiatric comorbidities in TLE patients, there are studies in patients with psychiatric disorders or epilepsy that evaluate gene methylation. Overall, the studies of DNA methylation in patients with depressive disorders frequently show significant associations, but no consistent changes have been reported, either for direction or position (Li et al., 2019; Penner-Goeke and Binder, 2019). Bellow we are revising some of these publications.

Several studies have reported that higher levels of DNA methylation in the *BDNF* gene are related to bipolar disorder, schizophrenia, borderline personality disorder, and depression (Perroud et al., 2008, 2013; Keller et al., 2010; Fuchikami et al., 2011; D’Addario et al., 2012; Kordi-Tamandani et al., 2012; Ikegame et al., 2013; Ferrer et al., 2019). Ikegame et al. (2013), by using high-resolution melt analysis, found that hypermethylation at promoters I and IV was also detected in patients with borderline personality disorder. There was a significant positive association between *BDNF* methylation levels and depression severity, impulsivity and child trauma, however no association was found between BDNF protein levels and DNA methylation levels (Ikegame et al., 2013).

Parrish et al. (2013) investigated whether DNA methylation contributes to *Grin2b* and *BDNF* expression during the epileptogenic process triggered by *status epilepticus (SE)*. They found that SE triggered increases in DNA methylation levels at the Grin2b/Nr2b promoter and decreased DNA methylation levels at the *BDNF* promoter, with a positive correlation of Grin2b/Nr2b and *BDNF* gene and protein expression levels in the epileptic hippocampus. They postulated that DNA methylation may be an early event triggered by SE that persists late into the epileptic hippocampus to contribute to gene expression changes in TLE (Parrish et al., 2013).

*SLC6A4* methylation was previously found to be associated with psychiatric disorders such as depression and alcoholism (Philibert et al., 2008; Devlin et al., 2010) and has been used as a peripheral marker for various neuropsychiatric disorders that involve 5-HT alterations (Hernández et al., 2002; Barkan et al., 2006; Marazziti et al., 2006). Kim et al. (2013) found that higher *SLC6A4* promoter methylation status was independently associated with post stroke depression at 2 weeks and more prominently at 1 year after the event, and was significantly associated with the worsening of depressive symptoms over 1 year. These findings were significant only in the presence of the 5-HTTLPR ss genotype.

A study conducted by Lei et al. (2015) evaluated environmental conditions and depressive symptoms. They perceived that higher levels of neighborhood crime, which is a stress factor, were associated with greater levels of self-reported depressive symptoms, and that this association was mediated by *SLC6A4* promoter methylation in individuals carrying the short allele for *SLC6A4*. These findings suggest that allele variants of *SLC6A4* may interact with environmental stressors to predict depressive symptoms in a genotype-dependent manner. Previously, Philibert et al. (2008), using samples from the Iowa Adoption Studies cohort, provided evidence that increased methylation levels in CpG islands overlapping with the transcriptional start site of *SLC6A4* was associated with decreased levels of mRNA, and those with the 5-HTTLPR s allele showed a trend toward higher methylation levels across CpG sites located in this upstream island. We were able to detect only three patients with methylated status in *SLC6A4*. This small number of patients precludes any further conclusions regarding a role of *SLC6A4* methylation in the genesis of neuropsychiatric comorbidities in patients with TLE.

The BDNF promoter region methylation and depression severity and treatment outcomes were the subjects of a recent editorial in Epigenomics (Reynolds, 2021), in which different studies, as early as 2013, indicated a role of epigenetic regulation associated with early life adverse events that predict different mood disorders later in life. Park et al. (2019), in a systematic review, claimed that stress-associated epigenetic changes in several genes, including *SLC6A4* and *BDNF*, were correlated with depression. Liu et al. (2021) demonstrated that spared nerve injury (which induces chronic pain) upregulated DNMTs and downregulated *BDNF* exon I expression in the hippocampus, leading to depressed behavior in rats. Blocking the upregulation of DNA methyltransferases (DNMTs) alleviated chronic pain-induced depression by up-regulating the expression of *BDNF* exon I. Moreover, they showed that ketamine alleviated depression symptoms, which was associated with normalization of DNMT levels and BDNF expression.

Xing et al. (2021) evaluated the methylation level of *BDNF* exon I in blood samples of patients diagnosed with MDD versus healthy controls. They found that there was a significant difference in the methylation level of *BDNF* exon I in MDD patients when compared to the control group, and that *BDNF* exon I methylation status could be used to distinguish depressed patients from their healthy counterparts. It is important to note that, in this study, methylation profile was not affected by use of a selective serotonin reuptake inhibitor (sertraline), nor was it associated with sertraline treatment success as monotherapy. However this differs from what Li et al. (2021) published recently in a study of 291 patients diagnosed with MDD and 100 healthy controls. Here, it was reported that methylation of *BDNF* exon VI was associated with MDD and antidepressant-induced remission in females, but not in males. The findings of both studies suggest that methylation at different promoter regions of the *BDNF* gene are implicated in MDD – however, one study evaluated exon I, whereas the other evaluated exon IV, which suggests that perhaps drug-induced methylation changes in different exons may or may not correlate with disease remission and/or response to treatment.

Still addressing drug response and methylation, Zhou et al. (2021) recently published a systematic review to evaluate the reproducibility of published changes of drug-induced DNA methylation in schizophrenia, bipolar disorder and MDD. They evaluated medication-induced DNA methylation changes, the relationship between DNA methylation and clinical improvement, and DNA methylation status across different medications. They concluded that only *BDNF* was consistent with DNA methylation changes in MDD, and that it was positively correlated with clinical improvement in MDD.

It is important to note that our study has limitations. First, measurement of methylation levels is semi-quantitative by its nature, and thus requires validation by other methods. We validated our findings using Sanger’s sequencing according to HRM protocol and found smaller variations in general methylation. Second, results from peripheral blood leucocytes may not directly be extrapolated to the human brain. However, Ikegame et al. (2013) noted that DNA methylation levels of four CpG sites in the *BDNF* gene promoter IV were very comparable between brain and blood tissues, which seems to indicate the plausibility of using peripheral blood in these analyzes. Moreover, the most commonly used source of DNA in *SLC6A4* methylation studies is blood tissue. Finally, the relatively small sample size of our study and the low methylation levels that we observed limited our statistical analysis. Nonetheless, our study might be useful for planning further studies aiming to evaluate a plausible role of epigenetics in the development of neuropsychiatric comorbidities in epilepsy.

## Conclusion

Concluding, we observed a significant association between lower levels of methylation the *BDNF* and *SLC6A4* gene promoters and presence of MDD or life history of depression in patients with TLE. However, studies with larger cohorts are necessary in order to fully elucidate whether methylation status correlates with mood disorders or other psychiatric comorbidities in epilepsy.

## Data Availability Statement

The raw data supporting the conclusions of this article will be made available by the authors, without undue reservation.

## Author Contributions

ICB, ALA, SL-S, MMB, and JWJB: conception and design of the research. ICB, LG, ICW, TLS, and JAB: acquisition of data. ICB, ALA, RB, JWJB, SL-S, and MMB: analysis and interpretation of results. ICB, TLS, RB, JWJB, SL-S, and MMB: drafting the work and revising the manuscript. All authors contributed to the article and approved the submitted version.

## Conflict of Interest

The authors declare that the research was conducted in the absence of any commercial or financial relationships that could be construed as a potential conflict of interest.

## Publisher’s Note

All claims expressed in this article are solely those of the authors and do not necessarily represent those of their affiliated organizations, or those of the publisher, the editors and the reviewers. Any product that may be evaluated in this article, or claim that may be made by its manufacturer, is not guaranteed or endorsed by the publisher.
